# Prevalence, Serotype Distribution, and Antimicrobial Resistance of *Streptococcus agalactiae* Among Pregnant Women in Greece: A Retrospective Study

**DOI:** 10.3390/jcm15166458

**Published:** 2026-08-20

**Authors:** Anthia Chasiakou, George Kaparos, Stamatia Chasiakou, Stiliani Demeridou, Vasiliki Koumaki, Athanasios Tsakris

**Affiliations:** 1 Department of Biopathology, Aretaieion University Hospital, National and Kapodistrian University of Athens, 76 Vassilisis Sofias Avenue, 11528 Athens, Greece; captenkap@yahoo.gr (G.K.); schasiakou@gmail.com (S.C.); demeridou@yahoo.gr (S.D.); 2 Department of Microbiology, Medical School, National and Kapodistrian University of Athens, 75 Mikras Asias Street, 11527 Athens, Greece; vkoumaki@med.uoa.gr

**Keywords:** *Streptococcus agalactiae*, GBS, pregnancy, serotypes, antimicrobial resistance, MLS_B_ phenotypes

## Abstract

**Background:** *Streptococcus agalactiae* (group B *Streptococcus*, GBS) remains a leading cause of invasive infections in pregnant women, fetuses, and neonates. Universal screening at 36–37 weeks of gestation and intrapartum antimicrobial prophylaxis are essential to prevent adverse outcomes. This study aimed to determine the prevalence, serotype distribution, and antimicrobial susceptibility of GBS isolates among pregnant women in Greece. **Methods**: Vaginal and rectal swabs were collected from pregnant women undergoing GBS screening between January 2021 and December 2025. Samples were processed using Todd-Hewitt broth with colistin and nalidixic acid, and cultured on blood agar and chromogenic media. The VITEK2 system was used for GBS identification and antimicrobial susceptibility testing against penicillin, erythromycin, clindamycin, tetracycline, levofloxacin, and vancomycin, according to EUCAST guidelines. The double-disk diffusion test was used for macrolide-lincosamide-streptogramin B (MLS_B_) phenotyping. Serotyping for all ten serotypes was conducted using a commercial latex agglutination assay. **Results**: Among 941 women screened, 118 (12.5%) were colonized with GBS. The most prevalent serotypes were III (29.7%), V (18.6%), Ib (14.4%), IX (10.2%), Ia (9.3%), and II (9.3%). All isolates were susceptible to penicillin. Resistance to erythromycin and/or clindamycin was detected in 37 isolates. In these isolates, the predominant MLS_B_ phenotype was constitutive (cMLS_B_, 78.4%), followed by inducible (iMLS_B_, 13.5%), L (5.4%), and M (2.7%) phenotypes. **Conclusions**: GBS colonization was detected in 12.5% of pregnant women, with serotype III predominating, underscoring its clinical relevance due to its association with invasive neonatal disease. Although penicillin remains fully effective, the observed resistance to macrolides and lincosamides, primarily mediated by the cMLS_B_ phenotype, raises concerns regarding alternative therapies.

## 1. Introduction

*Streptococcus agalactiae* (Lancefield group B streptococcus, GBS) is a commensal bacterium colonizing the gastrointestinal and genitourinary tracts. It is a leading cause of morbidity and mortality in neonates and young infants worldwide [1,2,3]. To reduce neonatal disease, the American College of Obstetricians and Gynecologists recommends universal culture-based screening of pregnant women using vaginal and rectal samples collected between 36 0/7 and 37 6/7 weeks of gestation, followed by intrapartum antimicrobial prophylaxis for colonized individuals [4].

Despite these measures, GBS remains a significant clinical concern worldwide. The global prevalence of maternal GBS colonization is estimated at approximately 18%, with regional variation ranging from 15.2% to 20.8% in Europe, 22% in North America, 11% in Asia, and 18.2% in Africa [5]. Vertical transmission occurs in nearly 50% of colonized mothers; however, invasive disease develops in only 1–2% of exposed neonates [4]. Transmission rates and disease burden vary across geographic regions, highlighting the importance of local epidemiological data.

Penicillin remains the first-line agent for the prevention and treatment of GBS infections, with sustained susceptibility reported globally. However, increasing resistance to alternative agents, particularly erythromycin and clindamycin, is of growing concern [6]. These antibiotics are commonly used in penicillin-allergic patients and, although chemically different, share overlapping mechanisms of action against Gram-positive organisms. Resistance to these antimicrobials can be expressed as resistance to 14-(erythromycin) and 15-(azithromycin) membered macrolides only (M phenotype), resistance to lincosamides (clindamycin) only (L phenotype), while cross-resistance to macrolides, lincosamides and streptogramin B, identified as MLS_B_ phenotype, can be constitutive (cMLS_B_) or inducible (iMLS_B_) [7]. Resistance to macrolides and lincosamides in antimicrobials can occur via different mechanisms, including ribosomal modification (target site alteration mediated by *erm* genes), active removal of the antimicrobial from the bacterial cell through efflux pumps (mediated by *mef* genes), and drug inactivation (enzymatic modification of the antimicrobial mediated by *Inu* genes) [7]. However, the dominant mechanism of resistance, so far, is ribosomal alteration through methylase enzymes encoded by *erm* genes. This modification confers cross-resistance, resulting in the MLS_B_ phenotype, and is attributed mostly to the expression of the *erm(B)* gene for cMLS_B_ and *erm(TR)*, a subclass of the *erm(A)* gene, for iMLS_B_ [8,9]. Resistance through efflux pumps is restricted to 14- and 15-membered macrolides, leaving clindamycin effective (M phenotype), mostly mediated by the *mef(A)* gene. Furthermore, exclusive resistance to clindamycin (L phenotype) is commonly mediated by active efflux encoded by *Isa* genes [8,9]. Interestingly, all resistance genes are linked to mobile genetic elements (integrative and conjugative elements or plasmids), often associated with resistance genes for other antimicrobials, mostly tetracyclines and aminoglycosides, thus facilitating horizontal transfer among bacteria [8]. Monitoring these resistance patterns is essential for guiding appropriate clinical management and prevention strategies for GBS infection.

Although screening and intrapartum antimicrobial prophylaxis have significantly reduced early-onset GBS disease, the emergence of antimicrobial resistance and the limitations of preventative antimicrobials have renewed interest in alternative preventive strategies, particularly maternal vaccination, which remains an unmet public health need [10]. In this context, characterization of circulating GBS serotypes is critical for vaccine development and implementation.

GBS is classified into ten serotypes (Ia, Ib, II–IX) based on capsular polysaccharide composition [2,11]. Among these, serotypes Ia, Ib, II, III, and V account for the vast majority of isolates from colonized pregnant women and invasive neonatal disease [5,12]. Notably, certain serotypes, particularly serotype III, are associated with hypervirulent clones and increased disease severity [13,14].

Epidemiological studies characterizing local GBS isolates in Greece are scarce. Therefore, this study aimed to: (1) assess the GBS rectovaginal colonization rate in pregnant women; (2) determine serotype distribution; (3) evaluate antimicrobial susceptibility profiles; and (4) examine the mechanisms of resistance to macrolide, lincosamide, and streptogramin B, to highlight the increasing resistance to these groups of antimicrobials, if any.

## 2. Materials and Methods

### 2.1. Patients, Specimen Collection and Processing

We included pregnant women aged 18 years or older, screened for GBS colonization (by both vaginal and rectal samples) at 36 0/7–37 6/7 weeks of gestation, between January 2021 and December 2025 at a maternity university hospital in Athens, Greece. Pregnant women younger than 18 years, presenting at less than 36 weeks of gestation, or patients for whom one of the samples (vaginal or rectal) was not available, were excluded from the study. After collection, vaginal and rectal samples were placed in Stuart’s bacterial transport medium and immediately sent to the laboratory for processing. Specimens were inoculated into Todd Hewitt broth supplemented with 15 μg/mL nalidixic acid and 10 μg/mL colistin (bioMérieux SA, Marcy-l’Etoile, France) and incubated at 37 °C for 24 h. After the 24 h incubation, 10 μL of broth was subcultured onto 5% sheep blood agar (Bioprepare, Keratea-Attiki, Greece) and chromogenic culture medium Liofilchem^®^ Chromatic™ Strepto B (Liofilchem^®^, Roseto d’Abruzzi, Italy), followed by an additional 24 h incubation. Culture plates were examined daily for two days for hemolytic and non-hemolytic colonies.

### 2.2. GBS Identification and Antimicrobial Susceptibility Testing

Suspected colonies were identified as GBS based on colony morphology, Gram staining, catalase test, bile-esculin test, and confirmed by the Christie-Atkins-Munch-Petersen (CAMP) test and with the rapid identification system I-dOne (Alifax S.r.l, Polverara, Italy). The I-dOne system (version 2.2.1) is the first CE-IVD-marked, reagent-free software using ATR-FTIR (Attenuated Total Reflection—Fourier Transform Infrared) spectroscopy. The identification of the pathogen is performed in about 2–3 min, directly from the colonies of primary culture, through specific spectra, compared with available databases of microorganisms, when defined threshold values are met [15]. The definitive identification and antimicrobial susceptibility testing were carried out using the automated system VITEK2 (bioMérieux, Marcy l’Etoile, France), according to the European Committee on Antimicrobial Susceptibility Testing (EUCAST) guidelines [16].

Resistance to erythromycin and clindamycin was further tested to confirm resistance phenotypes with the standardized double-disk diffusion test (D-zone test) using erythromycin (15 μg) and clindamycin (2 μg) disks placed 12–15 mm apart edge to edge, in accordance with the EUCAST guidelines [16]. Phenotypes were identified as: M when intermediate susceptibility or resistance to erythromycin and susceptibility to clindamycin was detected, L phenotype when intermediate susceptibility or resistance to clindamycin and susceptibility to erythromycin was observed, cMLS_B_ (constitutive mechanism of resistance to macrolides, lincosamides and streptogramin B) phenotype when resistance to erythromycin and either resistance or intermediate susceptibility to clindamycin were determined and, finally, iMLS_B_ (inducible resistance to macrolides, lincosamides and streptogramin B) phenotype when intermediate susceptibility or resistance to erythromycin and flattening of the zone of inhibition around clindamycin proximal to the erythromycin disk (D shape halo) was visible [7]. Reference strains *Streptococcus agalactiae* ATCC^®^ 13813™ and *Streptococcus agalactiae* ATCC^®^ 27956™ were used as controls.

### 2.3. GBS Serotype Identification

Serotyping was performed with a latex agglutination assay targeting capsular polysaccharides of all ten known (Ia, Ib, II–IX) *Streptococcus agalactiae* serotypes (Immulex™ *Streptococcus*-B Kit (SSI Diagnostica A/S, Hillerød, Denmark). The test is characterized by 100% (95% CI, 92–100%) sensitivity, 100% (95% CI, 98–100%) specificity, and 100% (95% CI, 99–100%) repeatability, while the reproducibility within the different groups of antisera and all antisera combined was 100% (95% CI, 99.4–100%). As the reference strain and positive control, the CultiControl™ *Streptococcus agalactiae* ATCC^®^ 13813™ (Liofilchem srl, Roseto d’Abruzzi, Italy) was used.

### 2.4. Statistical Analysis

Statistical analysis was performed using SPSS version 22.0 (IBM Corp., Chicago, IL, USA). Descriptive statistics were used to summarize the data and were presented as frequencies and percentages. Cross-tabulation analyses were performed to examine associations between categorical variables, and Pearson’s chi-square or Fisher’s exact test was applied to the cross-tabulation. Odds ratios (ORs) with corresponding 95% confidence intervals (95% CI) were calculated from 2 × 2 contingency tables using the Risk Estimate procedure in SPSS. Ninety-five percent confidence intervals for proportions were calculated using the Wilson score method without continuity correction. A two-sided *p*-value < 0.05 was considered statistically significant.

### 2.5. Ethical Approval

The study was approved by the Institutional Ethics Committee of Aretaieion University Hospital (no. 625/20 November 2024). Informed consent was waived as GBS data presented in this study were based only on tests routinely performed in our laboratory. All data were anonymized prior to analysis to ensure compliance with data protection and privacy regulations.

## 3. Results

### 3.1. Demographics and Maternal GBS Colonization Rate

A total of 941 pregnant women between 36 0/7 and 37 6/7 weeks of gestation, aged 22–45 years, were screened for GBS colonization during the study period. *S. agalactiae* was isolated from 118 women, corresponding to an overall colonization rate of 12.5% (95% CI: 10.6–14.8%).

### 3.2. Serotype Distribution

Serotype III was the most prevalent (35/118, 29.7%, 95% CI: 22.2–38.4%), followed by serotype V (22/118, 18.6%, 95% CI: 12.7–26.6%), Ib (17/118, 14.4%, 95% CI: 9.2–21.9%), IX (12/118, 10.2%, 95% CI: 5.9–16.9%), Ia (11/118, 9.3%, 95% CI: 5.3–15.9%), and II (11/118, 9.3%, 95% CI: 5.3–15.9%). Less frequently detected were serotypes IV (6/118, 5.1%, 95% CI: 2.4–10.7%), VI (3/118, 2.5%, 95% CI: 0.9–7.2%), and VIII (1/118, 0.9%, 95% CI: 0.2–4.6%). No isolates with unknown serotype were identified, while serotype VII was not detected.

### 3.3. Antimicrobial Susceptibility

The Minimal Inhibitory Concentrations (MICs), MIC_50_, MIC_90_, and MIC ranges of the tested antimicrobials for each GBS serotype are presented in Table 1.

All isolates were susceptible to penicillin and vancomycin. Resistance to erythromycin and clindamycin was detected in 29.7% and 25.4% of isolates, respectively, while resistance to tetracycline was markedly high (90.7%). Three (2.5%) GBS isolates exhibited resistance to levofloxacin, two serotype III and one serotype V (Table 2).

Among the 118 isolates, 37 (31.4%) exhibited resistance to erythromycin and/or clindamycin and were further characterized. Specifically, 29 isolates were resistant to both erythromycin and clindamycin, six to erythromycin only, and two to clindamycin only. The D-zone test revealed that 29 isolates (78.4%) expressed the constitutive MLS_B_ (cMLS_B_) phenotype, 5 (13.5%) the inducible MLS_B_ (iMLS_B_) phenotype, 2 (5.4%) the L phenotype, and 1 (2.7%) the M phenotype (Table 3). To evaluate the association between GBS serotypes and resistance to erythromycin and/or clindamycin, each serotype was compared with all remaining serotypes using odds ratios (ORs) and Pearson’s chi-square or Fisher’s exact test, as appropriate. Serotype III was significantly associated with resistance to erythromycin and/or clindamycin [x^2^ (1, n = 118) = 12.155, OR = 4.288, 95% CI: 1.841–9.987, *p* < 0.001], indicating significantly higher resistance rates compared with all other serotypes. No statistically significant associations were observed for the remaining serotypes, Ia, Ib, II, IV, V, VI, VIII, or IX (all *p* > 0.05).

This study covered the last two years of the COVID-19 pandemic in Greece (2021–2022) and the three years that followed (2023–2025). The distribution of GBS serotypes was compared between the COVID-19 pandemic that was still active in Greece during 2021–2022 and the post-COVID-19 pandemic period (2023–2025) using Pearson’s chi-square or Fisher’s exact test, as appropriate (Table 3). However, no statistically significant differences were observed for any serotype (all *p* > 0.05). Furthermore, resistance to erythromycin and/or clindamycin did not differ significantly between isolates collected during the pandemic period and those collected during the post-pandemic period (22.5% vs. 35.9%) [x^2^ (1, n = 118) = 2.205, *p* = 0.138].

## 4. Discussion

Our study spanned over the end of the COVID-19 pandemic in Greece (2021–2022) and a three-year post-pandemic period (2023–2025). Although the number of samples was lower in the pandemic period, probably due to social distancing guidelines and contagion fears, maternal colonization rates did not differ between the two periods. However, after the increase observed in 2023, the number of patients decreased in each following year, and this is likely due to the low birth rate Greece is facing in recent years. The general colonization rate was 12.5%, ranging from 11.7% to 13.5% during the five-year period. Previous reports from Greece recorded colonization rates ranging from 6.6% to 18.4% [17,18,19], although a low colonization rate of 1.7% has been recently documented in southern Greece [20]. Compared to global estimates, our data is similar to recent findings from Turkey reporting 11% colonization rate [21] and lower than the overall prevalence of 18% reported in a large meta-analysis [5], and data from Europe with rates between 15.6% in Serbia [22], 18.5% in Belgium [23], 21% in Portugal [24] and 29.1% in Denmark [25].

GBS serotyping is based on the capsular polysaccharide, a major virulence factor that facilitates immune evasion and persistence within the host [2,11,26]. To date, ten serotypes have been identified [27]. In our cohort, nine of the ten known serotypes were detected, with Ia, Ib, II, III, V, and IX being the most prevalent. This distribution aligns with global data, where serotypes Ia, Ib, II, III, and V account for approximately 98% of isolates [5] and are dominant in both Europe and North America [26].

Data on GBS serotype distribution in Greece remain limited. In our study, serotype III predominated, followed by V, Ib, IX, Ia, and II. This differs from earlier Greek reports in which serotypes II, III, Ia, and Ib were most common [17], suggesting temporal shifts in circulating strains. More recent findings from Greece and other European countries support the predominance of serotype III [28,29,30,31], although geographic variation persists, as illustrated by the high prevalence of serotype Ia in Bulgaria [32], serotype V in Poland [33] and Serbia [22], and serotype IX in Denmark [25]. Interestingly, no statistically significant differences were observed in the GBS serotypes identified during the COVID-19 pandemic and the post-pandemic period.

The predominance of serotype III is of particular concern due to its strong association with invasive neonatal disease. Serotypes Ia, Ib, II–V account for approximately 97% of invasive infections, with serotype III alone responsible for a substantial proportion of early- and late-onset disease, as well as the majority of meningitis cases [12,26,34,35]. In our study, nearly 30% of isolates belonged to serotype III, consistent with global trends [5], and this serotype was also associated with increased antimicrobial resistance.

Serotype IX, the most recently characterized serotype [11], was identified in 10.2% of isolates in our study. Although still underreported globally, similar rates have been described in London [30], while higher prevalence has been noted in Sweden [36] and Denmark [25]. Its implication in severe infections, including ultra-late-onset sepsis, further highlights its clinical relevance [37].

We did not detect serotype VII, consistent with recent reports from Europe [22,25,31], while serotypes VI and VIII were rare. This pattern is consistent with reports from Europe and North America, where these serotypes account for less than 5% of isolates, in contrast to significantly higher rates reported in Africa [38,39,40] and recently in Bulgaria [32].

Penicillin remains the first-line agent for GBS prophylaxis and treatment, with universal susceptibility reported over decades [4,6]. Our findings confirm continued susceptibility among all isolates, in agreement with previous studies from Greece [17,28]. Nevertheless, sporadic reports of reduced susceptibility underscore the need for ongoing surveillance [7,41].

In contrast, resistance to alternative agents, particularly macrolides and lincosamides, is increasing globally. In our study, resistance rates were 29.7% for erythromycin and 26.3% for clindamycin, reflecting a rising trend compared to earlier Greek data [17,28,42]. Similar variability has been reported internationally, with resistance rates differing widely across regions [33,38,43]. However, resistance rates to erythromycin and/or clindamycin did not differ between the isolates collected during the COVID-19 pandemic and those collected in the post-pandemic period. Phenotypic analysis revealed that the majority of resistant isolates exhibited the cMLS_B_ phenotype, consistent with previous findings [28,33]. These trends limit the reliability of macrolides and lincosamides as alternative therapies and reinforce current recommendations favoring other agents such as cefazolin or vancomycin [4]. The use of erythromycin and clindamycin provided useful therapeutic alternatives for colonized pregnant women allergic to penicillin. However, since they share similar binding sites, cross-resistance can be frequent. Obviously, when the cMLS_B_ phenotype is detected, these two antimicrobials cannot be used for prophylaxis, as they are clinically ineffective. The same is true for the iMLS_B_ phenotype, as well, where clindamycin resistance, although probably not apparent with a simple susceptibility test, is induced in the presence of erythromycin [7]. As a result, due to the increasing resistance rates to erythromycin and clindamycin, routine antimicrobial susceptibility testing for GBS isolates is imperative in penicillin-allergic women to prevent therapeutic failure. Moreover, the double-disk diffusion test must be performed to establish the exact resistance phenotype and further guide appropriate therapeutic management.

Tetracycline displayed the highest antimicrobial resistance rate (90.7%) among the GBS isolates, consistent with global reports and previous local data [22,28,32,33,38,43]. Tetracycline is a bacteriostatic antimicrobial, inhibiting protein synthesis and thus bacterial growth [6,7]. Three main mechanisms are responsible for tetracycline resistance: efflux pumps, ribosomal protection, and enzymatic inactivation [6,7,9]. More commonly, tetracycline resistance in GBS strains has been attributed to ribosomal protection proteins TetM and TetO (encoded by *tetM* and *tetO* genes) and efflux proteins TetK and TetL (encoded by *tetK* and *tetL* genes) [9]. Interestingly, co-resistance to tetracycline and macrolides has been documented [22] and has been linked to the insertion of *erm(B)* into the TN916 transposon carrying *tetM* [9,32], raising concerns about the rapid dissemination of macrolide resistance among GBS through the tetracycline-resistant isolates.

Levofloxacin is a broad-spectrum fluoroquinolone that inhibits bacterial replication by targeting enzymatic processes in DNA synthesis [7]. Fluoroquinolone resistance is increasing worldwide and is attributed to changes in topoisomerase IV (encoded by *parC* and *parE* genes) and DNA gyrase (encoded by *gyrA* and *gyrB* genes) [9]. We documented three isolates (2.5%) resistant to levofloxacin. Similar rates have been reported in Europe: in France, 1.5% [44] and 2.7% [45], 2.99% in Italy [46], or even higher, 4.6% in Sicily [31] and 10.28% in Bulgaria [47]. The increase in levofloxacin resistance is a matter of concern and is due to the extensive use of fluoroquinolones for genitourinary infections.

At present, the predominant prevention strategy against the wide spectrum of GBS-related disease is intrapartum antimicrobial prophylaxis. Its implementation has led to a significant reduction in many adverse outcomes. Prophylaxis through vaccination has always been a more efficient way to protect humanity against diseases. Despite continuous efforts, progress toward a safe and efficacious GBS vaccine has been slow. The two most advanced vaccine candidates that have undergone phase II trials are GBS6, a hexavalent conjugate vaccine developed by Pfizer, Inc. [10], and GBS-NN/NN2, a 2-component fusion-protein vaccine developed by Minervax [48]. GBS6 vaccine contains capsular polysaccharides from serotypes Ia, Ib, II, III, IV, and V, while the GBS-NN/NN2 vaccine contains surface proteins associated with GBS strains causing invasive disease [27,49]. Immunogenicity is a key factor for vaccine effectiveness. The GBS6 vaccine triggered an immune response to all serotypes in healthy pregnant women [50], while the GBS-NN/NN2 vaccine caused a robust immune response in healthy non-pregnant women [48].

Using a theoretical approach, our data show that the hexavalent vaccine could cover 86.4% of our study population. However, the weakness of such a vaccine relies on the potential of GBS capsular switching and on variations in serotype distribution among populations [51]. On the other hand, the surface proteins associated with the Minervax vaccine were documented in 99.3% of GBS isolates [51]; thus, this vaccine would probably cover a much larger population. More studies are warranted to elucidate GBS vaccine immunogenicity, as well as its safety and tolerability amongst diverse populations [26]. Interestingly, a recent study assessed, for the first time in Greece, pregnant women’s knowledge regarding GBS and their intention to receive a future GBS vaccine [52]. While general awareness of GBS was moderate (68.9%), intention to progress to GBS vaccination, when available, was detected in half (49.9%) of the participants. Significantly positive factors associated with increased acceptance were higher educational level, awareness of preventive measures, pregnancy complications, and prior pertussis vaccination during current pregnancy. In contrast, concerns about long-term adverse effects, impact on breastfeeding, and conception through assisted reproductive techniques were associated with reduced vaccination acceptance [52]. However, intention to accept a future GBS vaccine may not ultimately translate into actual vaccination, and other factors such as healthcare coverage or affordable cost may interfere in decision-making. As no official national GBS vaccine policy is available yet, and Greek women showed divided intent regarding future vaccine acceptance, healthcare providers must build the necessary trust to bridge knowledge gaps and safety concerns through effective communication strategies and adequate counseling prior to and during pregnancy.

This study has several limitations that must be addressed. The relatively small sample size and single-center design may not adequately represent colonization patterns in the general Greek population. Significant personal information regarding the study participants and possible risk factors (antimicrobial treatment before specimen collection, obstetric history, previous GBS colonization, intrapartum antimicrobial prophylaxis administered, neonatal colonization and disease) was not available. The lack of detailed clinical and epidemiological data limited the ability to control for confounding variables that potentially alter GBS colonization susceptibility and pathogenicity and hinder accurate assessment of antimicrobial exposure impact on resistance patterns. Additionally, molecular-based tests were not performed due to limited financial resources. Latex agglutination tests are rapid and inexpensive; however, they lack the accuracy and deep genetic potential of molecular genotyping. The analysis of resistance genes could have offered the needed explanation for the increased tetracycline resistance rate, the increasing erythromycin and clindamycin resistance, and the emergence of levofloxacin resistance. Finally, documenting the DNA sequence types and clonal complexes through multi-locus sequence typing and whole-genome sequencing could have provided an in-depth genetic analysis of the circulating GBS strains in Greece. Larger, multicenter studies are needed to better characterize the epidemiology of GBS colonization, serotype dynamics, genetic variations, and resistance patterns in Greece.

## 5. Conclusions

GBS remains a significant colonizer of pregnant women worldwide, with notable geographic variability in prevalence and serotype distribution. In this study, the colonization rate was considerable, and serotype III was the predominant type, reinforcing its clinical importance due to its association with invasive neonatal disease. Increasing resistance to erythromycin and clindamycin observed in our isolates is consistent with global trends and raises concerns regarding the effectiveness of alternative therapies. These findings highlight the importance of continued surveillance of antimicrobial susceptibility patterns and adherence to current guidelines favoring penicillin where appropriate. Ongoing monitoring of circulating GBS strains is essential for optimizing prophylactic and therapeutic strategies. Furthermore, detailed knowledge of serotype distribution and surveillance for emerging serotypes or antigenic variants will be critical for the development and implementation of future safe and adequate vaccines.

## Figures and Tables

**Table 1:** MIC (mg/L) distribution of tested antimicrobials in isolated GBS serotypes *.

Antimicrobial	Ia n = 11	Ib n = 17	II n = 11	III n = 35	IV n = 6	V n = 22	VI n = 3	VIII n = 1	IX n = 12	Total n = 118
Penicillin										
MIC_50_	≤0.06	≤0.06	≤0.06	≤0.06	≤0.06	≤0.06	≤0.06	-	≤0.06	≤0.06
MIC_90_	≤0.06	≤0.06	≤0.06	0.12	≤0.06	0.12	≤0.06	-	≤0.06	0.12
MIC range	≤0.06–0.12	≤0.06	≤0.06–0.12	≤0.06–0.12	≤0.06	≤0.06–0.12	≤0.06	≤0.06	≤0.06–0.12	≤0.06–0.12
Erythromycin										
MIC_50_	≤0.12	≤0.12	≤0.12	0.5	≤0.12	≤0.12	≤0.12	-	≤0.12	≤0.12
MIC_90_	0.5	2	0.5	4	4	4	≤0.12	-	0.5	4
MIC range	≤0.12–4	≤0.12–4	≤0.12–4	≤0.12–4	≤0.12–4	≤0.12–4	≤0.12	4	≤0.12–4	≤0.12–4
Clindamycin										
MIC_50_	≤0.25	≤0.25	≤0.25	≤0.25	≤0.25	≤0.25	≤0.25	-	≤0.25	≤0.25
MIC_90_	≤0.25	>0.5	>0.5	>0.5	≤0.25	>0.5	≤0.25	-	>0.5	>0.5
MIC range	≤0.25–>0.5	≤0.25–>0.5	≤0.25–>0.5	≤0.25–>0.5	≤0.25	≤0.25–>0.5	≤0.25	>0.5	≤0.25–>0.5	≤0.25–>0.5
Tetracycline										
MIC_50_	8	8	8	8	8	8	8	-	8	8
MIC_90_	8	8	8	8	8	8	8	-	8	8
MIC range	1–8	1–8	1–8	1–8	8	1–8	8	8	1–8	1–8
Levofloxacin										
MIC_50_	0.25	0.25	0.25	0.25	0.25	0.25	0.25	-	0.25	0.25
MIC_90_	0.25	0.5	0.25	0.5	0.25	0.5	0.25	-	0.25	0.25
MIC range	0.25–0.5	0.25–0.5	0.25	0.25–4	0.25	0.25–4	0.25	0.5	0.25–0.5	0.25–4
Vancomycin										
MIC_50_	0.5	0.5	0.5	0.5	0.5	0.5	0.5	-	0.5	0.5
MIC_90_	0.5	0.5	0.5	0.5	0.5	0.5	0.5	-	0.5	0.5
MIC range	0.5–1	0.5–1	0.5–1	0.5–1	0.5	0.5–1	0.5	1	0.5–1	0.5–1

* Serotype VII was not detected.

**Table 2:** Antimicrobial resistance among identified GBS serotypes.

Antibiotics	Ia n = 11	Ib n = 17	II n = 11	III n = 35	IV n = 6	V n = 22	VI n = 3	VII n = 0	VIII n = 1	IX n = 12	Total n = 118
Penicillin	0	0	0	0	0	0	0	0	0	0	0 (0%)
Erythromycin	2	3	2	18	1	5	0	0	1	3	35 (29.7%)
Clindamycin	1	3	2	15	0	5	0	0	1	4	31 (26.3%)
Tetracycline	8	15	9	33	5	21	3	0	1	12	107 (90.7%)
Levofloxacin	0	0	0	2	0	1	0	0	0	0	3 (2.5%)
Vancomycin	0	0	0	0	0	0	0	0	0	0	0 (0%)

**Table 3:** Yearly distribution of positive samples and phenotypes of the 37 resistant GBS isolates.

Year	Total Samples	Positive Samples	cMLS_B_	iMLS_B_	M	L
2021	151	18 (11.9%)	3	0	0	0
2022	168	22 (13.1%)	5	1	0	0
2023	228	28 (12.3%)	8	1	0	0
2024	215	29 (13.5%)	8	1	0	2
2025	179	21 (11.7%)	5	2	1	0
Total	941	118 (12.5%)	29	5	1	2

## Data Availability

All data generated or analyzed during this study are available from the corresponding authors on reasonable request.

## Abbreviations

The following abbreviations are used in this manuscript:

ATR-FTIR	Attenuated Total Reflection-Fourier Transform Infrared spectroscopy
CAMP	Christie-Atkins-Munch-Petersen
CE-IVD	Conformité Européenne—In Vitro Diagnostic
EUCAST	European Committee on Antimicrobial Susceptibility Testing
GBS	Group B *Streptococcus*
MIC	Minimal Inhibitory Concentration
MLS_B_	Macrolide-lincosamide-streptogramin B
